# Epidemiology of two decades of invasive meningococcal disease in the Republic of Ireland: an analysis of national surveillance data on laboratory-confirmed cases from 1996 to 2016

**DOI:** 10.1017/S0950268819000396

**Published:** 2019-03-12

**Authors:** D. Bennett, P. O'Lorcain, S. Morgan, S. Cotter, M. Cafferkey, R. Cunney

**Affiliations:** 1 Irish Meningitis and Sepsis Reference Laboratory, Temple Street Children's University Hospital, Dublin, Ireland; 2 Health Protection Surveillance Centre, Dublin, Ireland; 3 Department of Microbiology, Royal College of Surgeons in Ireland, Dublin, Ireland; 4 Department of Clinical Microbiology, Temple Street Children's University Hospital, Dublin, Ireland

**Keywords:** Epidemiology, meningococcal disease, *Neisseria meningitides*, surveillance

## Abstract

We examined the epidemiology of invasive meningococcal disease (IMD) in the Republic of Ireland (ROI) between epidemiological year (EY) 1996/1997 and EY2015/2016. Over the 20 EYs, 3707 cases were reported with annual incidence rates per 100 000 peaking at 11.6 in EY1999/2000, decreasing significantly to 1.5 in EY2015/2016. The highest disease burden was in infants and children <5, whereas adults aged ⩾65 years experienced the highest case fatality ratio (CFR) of 15.7% but over the study period the median annual CFR remained low (4.4%). Meningococcal serogroup B (menB) dominated (78%), followed by menC (17%), menW (1%) and menY (1%). The incidence of menC IMD declined significantly in all age groups after menC vaccine introduction in 2000. MenB incidence also declined over the 20 EYs with decreasing trends in all age groups under 65, including an almost 50% decrease in infants over the final four EYs. IMD incidence in the ROI has declined, partly attributable to menC vaccination success, coupled with a spontaneous decline in menB. However, recent gradual increases in non-menB IMD and the introduction of vaccines targeting menB demand continued detailed surveillance to accurately monitor trends and to assess vaccine impact.

## Introduction

Invasive meningococcal disease (IMD), defined as acute and severe infection by the bacterium *Neisseria meningitidis*, results in substantial neurological morbidity and mortality worldwide [1]. Clinical presentations of IMD include meningitis, severe sepsis, septic shock and less commonly, pneumonia and arthritis. Meningococci are characterised according to polysaccharide capsule, a main determinant of virulence, and 13 serogroups have been identified. Six serogroups (A, B, C, W, Y and X) are responsible for the majority of IMD cases worldwide, although distribution varies by geographical region and time, with most areas experiencing major cyclical fluctuations in the incidence of endemic disease and the occurrence of outbreaks and epidemics [1].

Laboratory-based epidemiological surveillance is particularly important for monitoring changes in incidence and serogroup distribution over time. Accurate and detailed surveillance, combining clinical, epidemiological and laboratory data, is critical for the diagnosis and management of suspected IMD in the clinical setting, as well as informing national policy for the introduction of preventive measures such as vaccination and monitoring the impact of such interventions in the population. In the Republic of Ireland (ROI), national IMD surveillance is performed by the Health Protection Surveillance Centre (HPSC) and the Irish Meningitis and Sepsis Reference Laboratory (IMSRL). All forms of bacterial meningitis have been denoted as notifiable diseases in ROI since 1982. Then, prompted by an increase in the number of cases notified during the early 1990s, the IMSRL (originally named the Irish Meningococcal and Meningitis Reference Laboratory) was established in 1996 to provide a national service for the non-culture diagnosis of IMD using polymerase chain reaction (PCR) on specimens from normally sterile sites and species confirmation with serological and molecular epidemiological typing of *N. meningitidis* isolates. Enhanced surveillance was also introduced at that time. In 2004, IMD became a notifiable disease in its own right instituting a legal obligation to report all cases of IMD. Case definitions for IMD introduced by the Department of Health in 1997 were also formally adopted in 2004 and subsequently updated in January 2012 (http://www.hpsc.ie). Consequent of these measures, detailed IMD surveillance data for ROI are available since epidemiological year (EY; July 1 to June 30) 1996/1997.

Here we describe the epidemiology of IMD in the ROI for the 20 EY period between EY1996/1997 and EY2015/2016, spanning pre- and post-introduction of the meningococcal C conjugate (MCC) vaccine (October 2000), changes to its scheduling, the introduction of the smoking in public places ban in March 2004 (http://www.ntco.ie) and also the epidemiology of IMD prior to the introduction of the 4CMenB (Bexsero®) vaccine in December 2016.

## Methods

Records maintained by the IMSRL of all laboratory-confirmed IMD cases diagnosed in the ROI between EY1996/1997 and EY2015/2016 were examined to determine the disease burden, case demographics, method of diagnosis and distribution of meningococcal serogroups.

To verify the completeness of the IMSRL database, all records of confirmed cases, notified to the eight regional Departments of Public Health since 1999, held in the national integrated electronic Computerised Infectious Disease Reporting (CIDR) system since its establishment, were matched to corresponding IMSRL case records [2]. Fatal outcome as recorded by IMSRL and, where possible, validated with a CIDR notification for cases diagnosed since EY1999/2000 was examined to assess the case fatality ratio (%CFR) for IMD over the study period.

Population data used were taken from http://www.cso.ie/px/pxeirestat/Database/eirestat/Annual%20Population%20Estimates/Annual%20Population%20Estimates_statbank.asp?sp=Annual%20Population%20Estimates&Planguage=0&ProductID=DB_PE (Table PEA11: Population estimates from 1926 by Single Year of Age, Sex and Year; Table PEA01: Population Estimates (Persons in April) by Age Group, Sex and Year and Table PEA07: Estimated Population (Persons in April) by Age Group, Sex, Regional Authority Area and Year) on 1 June 2017. To correspond with EY (July 1 to June 30), population data were calculated by using 75% of Year 1 (July–March, 9 months) and 25% of Year 2 (April–June) data. Case incidence figures are reported in cases/100 000 population over an EY period, unless otherwise specified. Cumulative numbers for each age group over all EY were used for risk ratio calculations.

Statistical analysis was performed using MS-Excel, version 2010 (Microsoft Corp., Seattle, WA, USA) and Stata, version 14 (StataCorp LP, College Station, Texas, USA). The *χ*^2^ analysis was used to test for difference in proportions and analysis of trend examining for overall increase or decrease over the 20-year period was performed using the non-parametric Kendall's rank correlation coefficient test. Age as a numerical variable was compared across different meningococcal serogroups by calculating median and interquartile ranges (IQR), and comparing them using the Kruskal–Wallis test (available at http://www.mathcracker.com/kruskal-wallis.php). For all statistical analyses *P* values of <0.05 were considered significant.

## Results

### Disease incidence

**Fig. 1.**
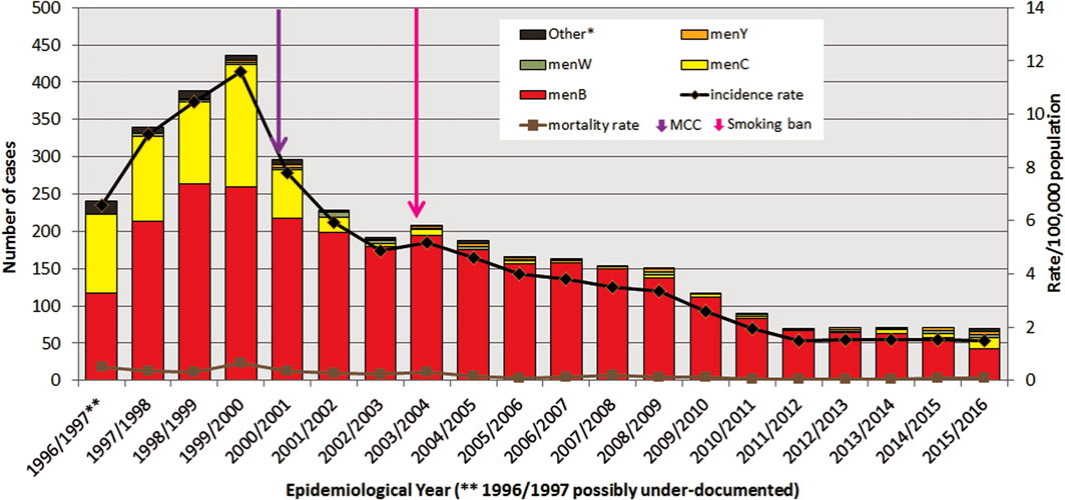
Number of laboratory-confirmed invasive meningococcal disease cases by meningococcal serogroup, and annual disease incidence and mortality rates per 100 000 population between epidemiological year (EY) 1996/1997 and EY2015/2016, in Republic of Ireland. EYs when the meningococcal C conjugate vaccine (MCC) was introduced into the national immunisation programme and the national ban on smoking in public places was introduced are indicated. *Other denotes non-serogroup B, C, W or Y meningococci (i.e. serogroups X, Z and serogroup non-identified). **Data for EY1996/1997 possibly incomplete as national non-culture diagnosis service rolled-out in November 1996 following establishment of IMSRL.

In the ROI between EY1996/1997 and EY2015/2016, there were 3707 laboratory-confirmed cases of IMD recorded in the IMSRL. These 3707 cases included records for all 2739 cases classified as confirmed reported to CIDR between EY1999/2000 and EY2015/2016 and 968 cases from between EY1996/1997 and EY1998/1999 that pre-date CIDR establishment. The annual incidence rates of IMD per 100 000 population (IR) demonstrated significant variation (Fig. 1); rising over the first three EYs to a peak incidence of 11.62 in EY1999/2000 (436 cases) which was initially followed by a sharp decline for the next three EYs to 4.88 in EY2002/2003 (192 cases) and subsequently by a more gradual decline before levelling off over the final five EYs to the lowest incidence observed over the study period of 1.49 in EY2015/2016 (70 cases; *P* < 0.0001; Table 1).

**Table 1.** Invasive meningococcal disease in Republic of Ireland during a 20 epidemiological year (EY) period from EY1996/1997 to EY2015/2016 by incidence rate per 100 000 population by meningococcal group, case age and by laboratory method of confirmation

Parameter/age group		Range	Median	95% CI	Kendall's score	*P* value
IMD incidence rate	Total	1.49–11.59	3.91	1.95–5.93	−166	**<0**.**0001**
	Under 1 year	25.13–187.55	69.60	39.02–115.70	−172	**<0.0001**
	1–4 years	3.67–71.65	23.04	9.09–34.32	−158	**<0.0001**
	5–9 years	1.45–17.43	5.00	1.87–7.46	−122	**0.0001**
	10–14 years	0.32–13.71	3.20	1.37–5.86	−138	**<0.0001**
	15–19 years	1.78–19.03	5.90	3.70–9.96	−124	**0.0001**
	20–24 years	0.31–7.19	2.20	1.08–3.52	−94	**0.0026**
	25–44 years	0.07–1.44	0.48	0.32–0.57	−116	**0.0002**
	45–64 years	0.09–1.78	0.59	0.44–0.93	−96	**0.0021**
	65+ years	0.36–1.89	0.81	0.67–1.16	−58	0.0644
MenB incidence rate	Total	0.89–7.08	3.54	1.82–4.85	−162	**<0.0001**
	Under 1 year	17.28–136.40	66.91	39.02–95.49	−160	**<0.0001**
	1–4 years	2.57–48.42	19.45	8.37–30.20	−150	**<0.0001**
	5–9 years	1.22–11.75	3.17	1.78–6.39	−100	**0.0013**
	10–14 years	0.32–7.18	2.88	1.32–3.56	−124	**0.0001**
	15–19 years	0.69–9.62	5.05	2.83–6.11	−88	**0.0048**
	20–24 years	0.31–3.51	1.58	0.91–2.67	−78	**0.0125**
	25–44 years	0.07–0.84	0.37	0.21–0.49	−98	**0.0016**
	45–64 years	0.00–1.07	0.38	0.13–0.76	−64	**0.0410**
	65+ years	0.00–1.44	0.60	0.40–0.71	−5	0.8967
MenC incidence rate	Total	0.02–4.37	0.12	0.07–0.52	−90	**0.0039**
	Under 1 year	0.00–37.89	0.00	0.00–5.46	−91	**0.0013**
	1–4 years	0.00–23.56	0.00	0.00–2.73	−82	**0.0029**
	5–9 years	0.00–9.27	0.15	0.00–0.64	−87	**0.0028**
	10–14 years	0.00–7.02	0.00	0.00–0.63	−70	**0.0111**
	15–19 years	0.00–9.52	0.35	0.00–1.71	−67	**0.0297**
	20–24 years	0.00–3.92	0.28	0.00–0.60	−118	**0.0001**
	25–44 years	0.00–0.48	0.07	0.00–0.17	−72	**0.0170**
	45–64 years	0.00–0.71	0.11	0.00–0.25	−79	**0.0102**
	65+ years	0.00–0.95	0.09	0.00–0.23	−57	0.0512
MenW incidence rate	Total	0.00–0.16	0.06	0.02–0.10	−31	0.3295
MenY incidence rate	Total	0.00–0.11	0.05	0.02–0.08	10	0.7693
Median case age	All IMD cases	1.97–13.59	2.95	2.53–3.33	−34	0.2843
	MenB IMD cases	1.70–7.36	2.63	2.19–2.92	−2	0.9741
	MenC IMD cases	4.36–22.23	10.80	5.70–20.33	18	0.0763
Laboratory confirmation	% Cases diagnosed by PCR only	38.33–71.01	62.20	58.44–66.18	16	0.6265
Ratio of culture diagnosed cases to all cases by serogroup	MenB	0.81–1.19	0.97	0.94–1.00	−43	0.1417
	MenC	0.00–3.45	1.07	1.00–2.01	24	0.4207
	MenW	0.00–3.09	1.68	0.00–2.41	−22	0.4546
	MenY	0.00–3.84	1.61	0.00–2.62	−6	0.8587

CI, confidence interval; IMD, invasive meningococcal disease; menB, meningococcal serogroup B; menC, meningococcal serogroup C; menW, meningococcal serogroup W; menY, meningococcal serogroup Y; PCR, polymerase chain reaction non-culture diagnosis.

*P* value of <0.05 (highlighted in bold text) denotes a significant trend over the 20 EY period, the direction and extent of which can be inferred by the positive (increasing) or negative (decreasing) Kendall's score value.

The general %CFR was low and stable; it ranged from 2.25% to 7.50% of cases, with a median of 4.36% (95% confidence interval (CI) 3.68–5.19) over the 20 EYs. Overall, the mortality rate per 100 000 population also remained low, ranging from 0.04 to 0.64 with a median of 0.16 (95% CI 0.09–0.32) deaths per 100 000 population recorded (Fig. 1).

### Case demographics

**Fig. 2.**
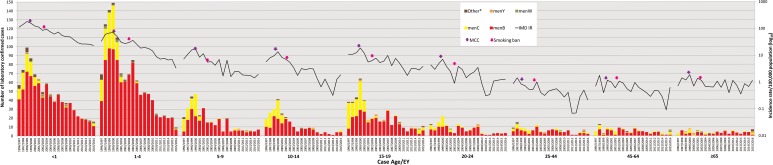
Laboratory-confirmed invasive meningococcal disease (IMD) by meningococcal group and incidence rate per 100 000 population by case age between epidemiological year (EY) 1996/1997 and EY2015/2016, in Republic of Ireland. EYs when the meningococcal C conjugate vaccine (MCC) was introduced into the national immunisation programme and the national ban on smoking in public places was introduced are indicated. Age was not known for 10 confirmed IMD cases. *Other denotes non-serogroup B, C, W or Y meningococci (i.e. serogroups X, Z and serogroup non-identified).

Age data were available for 3697 cases (99.7%). The serogroup distribution and incidence rates of IMD by age group for each EY are shown in Figure 2 and Table 1. Overall, the highest incidence was observed among children <1 year of age (total IR 74.27/100 000; range 187.55 in EY1998/1999 to 25.13 in EY2015/2016), among whom 927 (25%) of all IMD cases occurred (<1 year olds comprised approximately 1.5% of total national population over the study period; relative risk (RR) 22.2; 95% CI 20.6–23.9; *P* < 0.0001). Incidence was also high in children aged 1–4 years (total IR 25.70/100 000, range 71.65 in EY1999/2000 to 3.67 in EY2015/2016; *n* = 1245 (33.6%) cases overall) with another incidence peak in cases aged 15–19 years (total IR 7.35/100 000; *n* = 449 (12.1%) cases overall; Fig. 2). There was an over-representation of IMD cases in these age groups, as children aged 1–4 years and teenagers aged 15–19 years comprised 5.8% and 7.3% of the national population, respectively; reflected by the corresponding RRs of 8.3 (95% CI 7.75–8.89; *P* < 0.0001) and 1.76 (95% CI 1.6–1.9; *P* < 0.0001).

Over the study period, there was an increase in median case age; in EY1996/1997 the median case age was 4.1 years, whereas in EY2015/2016 the median age was 13.6 years, although the overall change in median age was not determined to be significant (Table 1). During the five EYs until EY2000/2001, a median of 94.25% of cases occurred in children and young adults <25 years (median IR 20.41); this dropped to 83.1% (median IR 3.77) in the latter five EYs since EY2011/2012. The decline in disease among those over 25 years was not as pronounced (median IR for EY1996/1997–EY2000/2001 of 0.79 falling to 0.39 in EY2011/2012–EY2015/2016).

Mortality rates mirrored incidence rates, with the highest among children aged <1 at 3.04/100 000 and lowest among 25–44 year olds at 0.012/100 000 over the study. However, %CFR was highest in patients aged ⩾65 years (15.7%; RR 3.73, 95% CI 2.25–6.19; *P* < 0.0001), although the incidence of IMD was one of the lowest in that age group (in total 0.92/100 000 population, range 1.89/100 000 in EY1999/2000 to 0.36/100 000 in EY2012/2013) compared with other age groups.

Gender data were available for 3702 (99.9%) cases and overall the male:female ratio was 1.18:1.0, with 54.2% of cases being male. Equivalence (1.0:1.0) gender ratios were observed in most age groups; except for in children <1 year with a male:female ratio of 1.35:1.0, in 1–4 year olds 1.26:1.0, in 15–19 year olds 1.21:1.0 and in those aged ⩾65 years 0.53:1.0. However, controlling for male:female ratios in the national population, the absolute male:female ratios were 1.29:1.0 in <1 year olds, 1.50:1.0 in 1–4 year olds, 1.15:1.0 in 15–19 year olds and 0.68:1.0 in the over 65 years age group, with the ratios for all other age groups remaining at or close to equivalency.

### Laboratory method of case confirmation

**Fig. 3.**
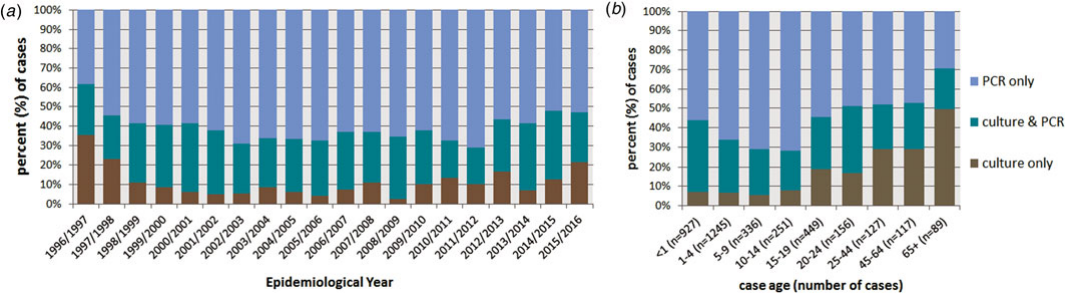
Invasive meningococcal disease (IMD) by method of laboratory confirmation (a) by epidemiological year (EY) and (b) according to case age for all laboratory-confirmed cases diagnosed in the Republic of Ireland between EY1996/1997 and EY2015/2016 (age was not known for 10 confirmed IMD cases).

All cases were confirmed by either sterile site PCR or culture or both. Of the 3707 cases, diagnosis of 2222 (59.9%) cases was by PCR only, ranging from 38.3% in EY1996/1997 to 71.1% in EY2011/2012; 427 (11.5%) cases were diagnosed by culture alone and 1058 (28.5%) by both culture and PCR (Fig. 3a). Overall there was no discernible change in the proportions of cases diagnosed by PCR only in each EY throughout the study period (Table 1). Differences were observed between method of case confirmation and case age (Fig. 3b); 70.8% (63/89) of cases aged ⩾65 years were diagnosed by culture (alone or combined with PCR) whereas only 28.3% (71/251) of cases aged 10–14 years yielded a meningococcal isolate. Cases aged ⩾20 years were more likely to be diagnosed by culture than cases aged <20 years (55.4% *vs.* 37.6%; *P* < 0.0001). Among the age groups with the highest incidence of IMD (infants and those aged 15–19 years), a meningococcal isolate was only recovered from 45% of cases.

### Serogroup breakdown and age-related incidences

Meningococcal serogroup was identified for 3645 (98.3%) of the 3707 cases (Fig. 1). The breakdown according to serogroup was 2908 (78%) due to serogroup B (menB), 642 (17%) to serogroup C (menC), with serogroup W (menW) and serogroup Y (menY) accounting for 1% each (*n* = 50 and 41, respectively). Three cases were due to serogroup X and one to serogroup Z, all diagnosed by culture only. The serogroup for 62 cases was not identified, the majority of which (*n* = 40; 74.2%) were associated with disease pre-EY2001/2000. Fifty-six (90%) of all of the non-grouped cases were diagnosed by PCR alone and 51 of these were diagnosed prior to the introduction of specific menW and menY PCR genogrouping assays [3]. There was no difference in the ratios of menB or menC cases diagnosed by PCR alone (or conversely by culture) compared with total IMD cases over the 20 EYs, so there was no bias due to method of confirmation (Table 1). Together menB and menC accounted for more than 90% of all cases in each EY until EY2014/2015 and EY2015/2016 when they accounted for 89% and 81.5% of all cases, respectively.

**Fig. 4.**
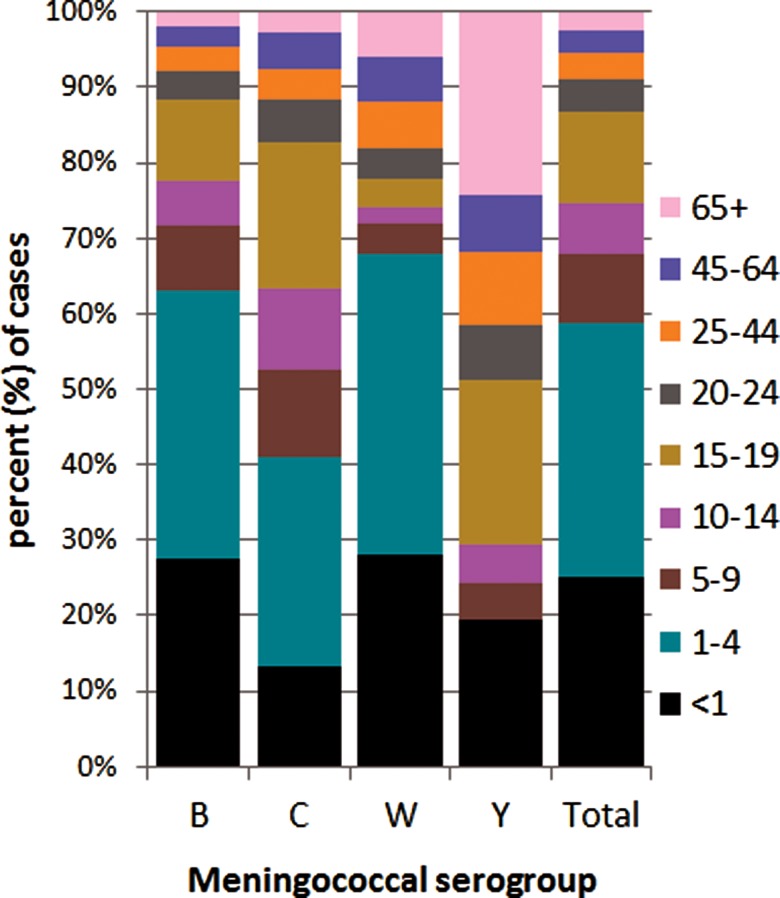
Invasive meningococcal disease cases by serogroup according to case age for all laboratory-confirmed cases diagnosed in the Republic of Ireland between EY1996/1997 and EY2015/2016 (age was not known for 10 confirmed IMD cases).

The distribution of meningococcal serogroup also varied with age of case, even considering the dominance of menB (Fig. 4). The median age was lowest for menW (1.9 years, IQR 16.4), higher in menB (2.6 years, IQR 12.2) and in menC (7.9 years, IQR 15.6) and highest for menY (18.1 years, IQR 52.8) (*P* = 0.0001). The IR for all serogroups was highest among the <1 year olds, although proportionally less menC disease was observed among this age group compared with other groups. The highest proportion of menB and menW disease was seen in the <5 year olds and the highest proportion of menC disease was seen in the 1–4 and 15–19 year olds. No menY disease was seen in the 1–4 year olds, but the ⩾65 year olds had the highest proportion of menY. Parallel case age patterns to those noted with overall case age were observed with menB and menC cases. Similarly, no differences were observed with gender of case and disease due to menB, menC and menY, although the male:female ratio for menW cases was 2.57:1.0, but total number of menW cases was small.

### Serogroup prevalence over time

The proportion of serogroups changed from year to year, though menB dominated all 20 EYs. In the initial four EYs, EY1996/1997 to EY1999/2000, the predominance of menB was less pronounced as menC IMD accounted for between 30% and 45% of cases. During that period, 493 menC cases were recorded or 76.8% of total menC IMD over the 20 EYs. MenC incidence rates peaked at 4.37 cases in EY1999/2000, immediately preceding MCC vaccine introduction (October 2000). Since then the incidence rate of menC IMD declined significantly to 1.68 in EY2000/2001 to a low of 0.02 in EY2012/2013; but with a slight increase to 0.32 in EY2015/2016 (*P* = 0.0039; Table 1).

MenB disease peaked in EY1998/1999 with an incidence rate of 7.1 (263 cases). There has been a gradual pattern of menB IMD decline since then, with steady year-on-year decreases in incidence rate observed (*P* < 0.0001; Table 1). In EY2015/2016, just 42 menB cases were identified with the lowest ever recorded menB incidence rate of 0.89/100 000 population. The decline in menB IMD was observed in all age groups and was significant in all age groups under 65 years (Table 1). The greatest decreases were seen among the <5 year olds and those aged 10–14 years. In the <1 year olds, a decrease in menB cases from 22 to 11 was observed between EY2011/2012 and EY2015/2016 corresponding to a drop in incidence rate from 30.2 to 16.3/100 000 population. The decline was even more pronounced among the 1–4 year olds where a decrease from 21 to seven cases was observed over the same time period, equating to a drop in incidence rate from 7.4 to 2.4/100 000 population.

MenW and menY disease each accounted for only 1% of cases over the 20 EY period. Actual annual numbers were small for both serogroups ranging from 0 to 6 for menW and 0 to 5 for menY, corresponding to highest incidence rates of 0.16/100 000 for menW in EY2001/2002 and of 0.11/100 000 population for menY in EY2008/2009. Neither exhibited a significant trend over the 20 EYs (Table 1) although since EY2013/2014 there has been a small but gradual rise in the proportions of cases due to menW and menY, but numbers remain low.

## Discussion

Over the past 20 EYs, there has been considerable variation in incidence rates of laboratory-confirmed IMD, in the ROI. The highest rate recorded was 11.6/100 000 in EY1999/2000, which has since gradually declined to the lowest ever recorded rate of 1.50/100 000 in EY2015/2016. The decline, largely attributed to the success of the MCC vaccine introduced in October 2000 in virtually eliminating menC disease, is also due to a spontaneous decrease in menB disease in the absence of any obvious intervention. Similar significant decreasing trends in menC IMD were observed in other countries that also implemented the MCC vaccine into their routine schedule compared with rates in countries that had no MCC vaccination policy [4]. This suggests the high uptake rate of the vaccine and the development of herd immunity among the unvaccinated cohorts [5] similar to the experience in the UK [6, 7].

Nonetheless, with the reduction in menC, disease due to menB predominated and accounted for the vast majority of cases in each EY after the introduction of the MCC in the ROI, as was the case in many EU countries [4], in particular those with MCC vaccination policies. MenB cases have in ROI however, also reduced significantly over the period with, in EY2015/2016 a sixfold drop in actual numbers since peak case figures in EY1999/2000 representing an eightfold drop in incidence rate. No specific anti-menB interventions were introduced to trigger this, although perhaps lifestyle changes including the introduction of the smoking ban from public places may have contributed to this decline [8]. Similar spontaneous gradual declines in menB IMD cases have also been observed in other European countries, also in the absence of any specific interventions [9–11]. Furthermore, despite early concerns of possible vaccine-mediated capsule switching or serogroup replacement, only 4.3% of all IMD diagnosed since MCC introduction was due to non-menB or menC. In addition, as is the case in most EU countries [1, 4], IMD due to menW and menY was rare in ROI throughout the 20 EYs. Unlike experiences in the UK and Sweden, where significant recent rises in rates of disease due to menW and menY have been observed [12–16], constant but low rates of menW and Y disease were observed here. Nevertheless in 2014, ROI still had one of the highest rates of IMD in Europe, and the highest rate among countries that have a routine MCC vaccination programme at 1.3/100 000 population [4, 17].

The slight male predominance among IMD cases in ROI has also been documented by several other studies [10, 11, 18–20] and which has been partly attributed to gender-related physiology and gender-specific behaviour [20–23]. Also, the distribution of case age profiles was similar to the age profiles found with IMD in other countries [1, 10, 11], with infants and young children experiencing the greatest burden of disease and a smaller secondary disease peak observed in older teenagers. However, an age-shift was discerned with an increase in median case age observed between EY1996/1997 and EY2015/2016. A similar increase in case age was also documented in The Netherlands [11] and those authors speculated that this was due to MCC vaccination and an ageing population overall. These explanations could also be appropriate for ROI. The MCC vaccine was rolled out as part of the infant immunisation schedule in 2000 with a catch-up campaign to include those up to 23 years of age. Overall levels of coverage were estimated to be 70%; highest in the <18 year olds at more than 80% [5]. The implementation of this vaccine resulted in the significant decline in menC IMD as a consequence of population herd immunity with the eradication of menC carriage among the principal carriers (older teenagers/young adults), thereby interrupting the transmission of menC [6, 7]. Furthermore, it is in this age group (<25 years) where the decline in IMD (menC, but largely menB) was most pronounced compared with the older age groups. The increase in the annual median case age values reflects this shift (the median age value is less influenced by the size of <25 age group and as a result the median age each year increases or the proportion of cases attributable to those aged <25 has declined much more sharply over time (compared with older age groups) and therefore, has resulted in a change/increase in the median case age). However, there were no discernible impacts due to menY or menW cases, which are traditionally associated with causing IMD in individuals aged over 65 years [9, 24], reflecting their long-standing low incidence rates in ROI.

Significant declines in menB disease rates were evident in all age groups except in ⩾65 year olds. Disease incidence due to menB (and other meningococcal serogroups) in this older population has, however, remained low. The greatest decreases were observed in children <5 years old and also in the 10–14 year olds, although these age groups still accounted for just over a quarter of all menB cases reported in EY2015/2016. The <1 year olds still had the highest menB incidence rate of any age group at 16.3/100 000 in EY2015/2016 supporting the introduction of the multi-component menB vaccine, 4CMenB, into the national infant immunisation programme in December 2016. However, it may be difficult to assess the impact of this vaccine in this age group due to low case numbers; the actual numbers of menB diagnosed in EY2015/2016 in <1 year olds (*n* = 11) was over 40% lower compared with the average number of menB cases diagnosed in this age group in the previous 4 years (18.7 cases). Furthermore, during the last four EYs, disease due to menY, menC and menW has increased in this age group (possibly due to spread from older populations), and accounted for 12.3% of all IMD in this age group in that period. 4CMenB is registered to prevent MenB disease [25] and its efficacy against meningococcal disease caused by other serogroups is unclear [26], although there may be some protection against menW [27, 28].

The use of PCR for the national non-culture diagnosis of IMD with determination of meningococcal serogroup has undoubtedly had a significant impact on the speed and accuracy of case confirmation. It has enabled not only improved individual case management, but also better public-health management through accurate evaluation of the impact of MCC vaccination and our understanding of meningococcal disease epidemiology. Over the 20 EY period, without PCR diagnosis in ROI, there would have been a large gap in the confirmation of disease and associated serogroup for almost 60% of cases from whom the causative meningococcal isolate was not cultured. PCR diagnosis of IMD is now an important fundamental tool in many reference laboratories and has made an invaluable contribution to the diagnosis of similar proportions of cases in many countries since its introduction [18, 29–31]. However, despite the advantages of case confirmation by PCR, there are also several limitations, not least in its inability to accurately monitor antimicrobial susceptibility and perform detailed meningococcal strain characterisation including examining antigen expression in the absence of an isolate. Although, whole genome sequencing (WGS) of pathogens directly from clinical material without culture is possible, process improvements are necessary to ensure success with all pathogen-positive material [32, 33]. Meanwhile, the lack of isolates recovered from cases within the age groups associated with the highest incidence of IMD in ROI is of grave concern as currently only limited epidemiological information can be obtained concerning the causative meningococcal strain from cases confirmed by PCR only. Also, given that meningococcal isolates were more likely to be recovered from cases aged ⩾20 years than from those aged ⩽19 years, our future understanding of meningococcal strain epidemiology could be biased.

Owing to these issues, it is imperative that every effort is made in an attempt to recover the causative organism associated with IMD, including correct sampling and rapid culturing of throat swabs. A recent study examining throat–blood meningococcal isolate pairs recovered from individuals with IMD using detailed characterisation by WGS found that they differed by only a very small number of genetic changes [34]. The importance of an isolate is even more crucial in view of the recent introduction of 4CMenB. 4CMenB is a multi-component protein-based vaccine with predicted incomplete strain coverage [27], and unlike traditional capsular-based meningococcal vaccines (e.g. MCC), its efficacy cannot be measured by examining meningococcal serogroup, alone. It is not a menB vaccine *per se* as other meningococcal serogroups possess many (and variants) of the components contained within the vaccine, as in fact do many of the non-pathogenic commensal *Neisseria* species [26, 27]. Indeed, following vaccination and exertion of immunologic pressure, it is reasonable to assume that strains not covered by 4CMenB or with incomplete coverage may emerge with variation occurring in strains once susceptible (those that harboured all or some of the exact vaccine components acquiring unmatched components from either other meningococci or from other *Neisseria* spp.) leading to the emergence of natural or acquired vaccine escape strains and ‘vaccine failure’ [35, 36]. Definitive and accurate evaluation of the impact of 4CMenB vaccination and monitoring of meningococcal epidemiology will only be achievable through detailed strain characterisation using WGS [36] and by determining levels of antigen expression, the latter is only possible with a viable meningococcal isolate [26].

In conclusion, important changes in the epidemiology of IMD have been observed in ROI during the last two decades. MenC IMD virtually disappeared following the implementation of a universal childhood immunisation programme effecting that most cases since then have been due to menB. Overall IMD incidence has decreased significantly and now remains relatively low, although the most affected cohorts are still infants and the very young with teenagers and adolescents also at an increased risk. Furthermore, given historical dynamics of IMD epidemiology (even over the past 20 EYs), it is unpredictable whether low disease incidence will persist and what potential regional or global impact emerging strains may have. In December 2016, 4CMenB (Bexsero®, GlaxoSmithKline, https://www.gsk.com) was introduced into the infant immunisation programme in Ireland to help mitigate this, but data regarding its overall efficacy are scant, especially in relation to the level and duration of protection offered, specific targeted strains and effect on carriage/herd immunity. Now more than ever, detailed surveillance is essential to monitor both the disease-associated and carried meningococcal populations circulating in ROI. WGS of meningococci allows the amassing of unprecedented levels of comprehensive and detailed information on a strain's genetic make-up, making it pivotal to the interpretation of meningococcal population biology dynamics and aid in the understanding of how the meningococcus can cause life-threatening invasive disease in some individuals and be just a harmless commensal in most others. From an epidemiological aspect, it is an invaluable tool that allows the exploration of capsule switching and, more specifically, the early detection of the emergence or re-emergence of hyperinvasive strains.

## Supplementary material

For supplementary material accompanying this paper visit https://doi.org/10.1017/S0950268819000396. click here to view supplementary material
