# Supplementary figures and images for: Epidemiology of two decades of invasive meningococcal disease in the Republic of Ireland: an analysis of national surveillance data on laboratory-confirmed cases from 1996 to 2016
Source: Epidemiol Infect. 2019 Mar 12;147:e142. doi: 10.1017/S0950268819000396 (PMC6518514; doi:10.1017/S0950268819000396)

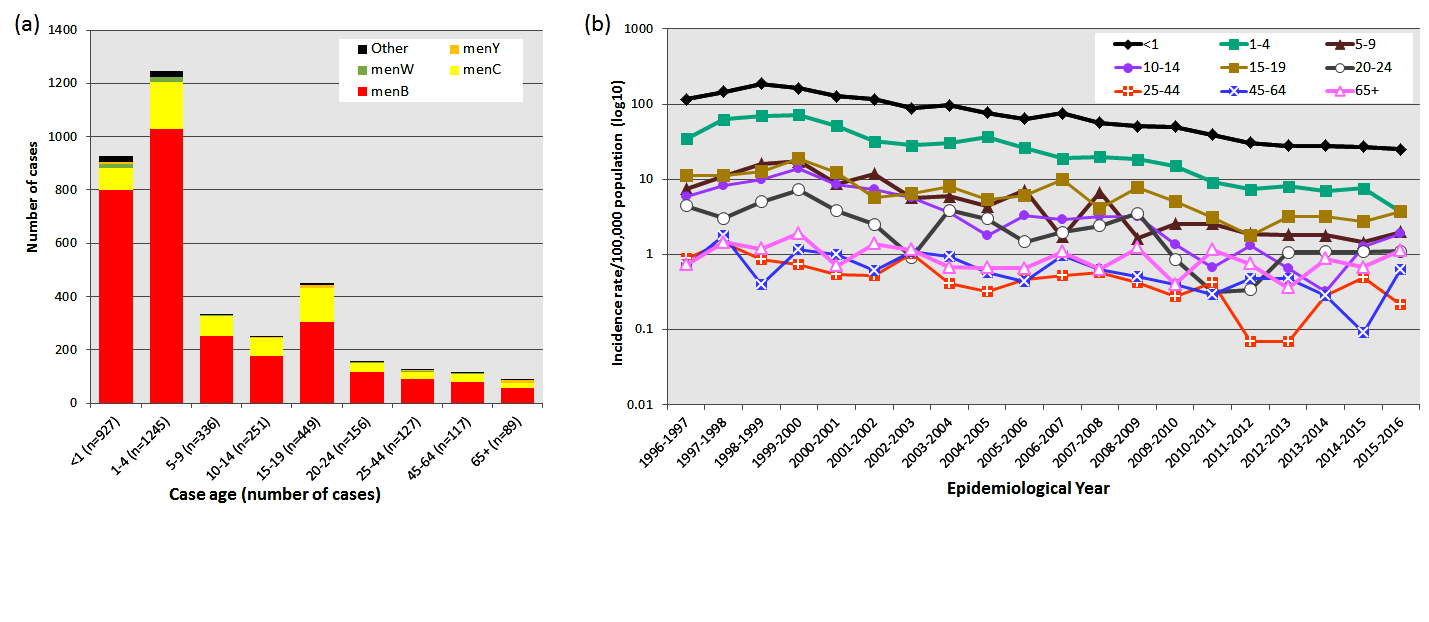

Supplement: Supplementary file 1 [file S0950268819000396sup001.tif]
